# Exploring Legume-Rhizobia Symbiotic Models for Waterlogging Tolerance

**DOI:** 10.3389/fpls.2019.00578

**Published:** 2019-05-08

**Authors:** Chiara Pucciariello, Alexandre Boscari, Andrea Tagliani, Renaud Brouquisse, Pierdomenico Perata

**Affiliations:** ^1^PlantLab, Institute of Life Sciences, Sant’Anna School of Advanced Studies, Pisa, Italy; ^2^Institut Sophia Agrobiotech, Centre National de la Recherche Scientifique, Institut National de la Recherche Agronomique, Université Côte d’Azur, Nice, France

**Keywords:** hypoxia, legumes, nitric oxide, oxygen sensing, symbiosis, waterlogging

## Abstract

Unexpected and increasingly frequent extreme precipitation events result in soil flooding or waterlogging. Legumes have the capacity to establish a symbiotic relationship with endosymbiotic atmospheric dinitrogen-fixing rhizobia, thus contributing to natural nitrogen soil enrichment and reducing the need for chemical fertilization. The impact of waterlogging on nitrogen fixation and legume productivity needs to be considered for crop improvement. This review focuses on the legumes-rhizobia symbiotic models. We aim to summarize the mechanisms underlying symbiosis establishment, nodule development and functioning under waterlogging. The mechanisms of oxygen sensing of the host plant and symbiotic partner are considered in view of recent scientific advances.

## Introduction

Global population is expected to reach around 9.6 billion in 2050 (Gerland et al., 2014), leading to a rise in the demand for food. Food issues are also aggravated by unexpected and increasingly frequent extreme weather events connected to climate change such as soil flooding or waterlogging, occurring especially in areas close to watercourses, characterized by poor soil drainage or exposure to monsoons.

In agriculture, the conversion to alternative, more ecologically sustainable sources is moving toward productive systems that reduce the input of fertilizers. Nitrogen (N) is one of the most important nutrients for crops and today a reduction in crop dependence on chemical N fertilization is essential. This is due to the cascade of environmental changes resulted from the huge increase of ammonia (NH_3_) production in the last century, such as water and soil pollution (Erisman et al., 2008). Legumes are well known for their agronomical and food properties, thanks to their capacity to establish a symbiotic relationship with endosymbiotic atmospheric dinitrogen (N_2_)-fixing rhizobia, thus contributing to natural N soil enrichment and reduced need for chemical fertilization. These crops are also a key protein resource for human and animal foods.

In legume plant roots, the interaction with rhizobia leads to the development of the nodule organ, where the nitrogenase enzyme reduces atmospheric N_2_ to NH_3_ which is afterward transferred to and assimilated by the plant. In parallel, the plant provides steady carbon source to the symbiont and a suitable microenvironment for development (Markmann and Parniske, 2009). When selecting stress-tolerant legume crops, the impact of soil flooding and waterlogging on N_2_ fixation and legume productivity need to be considered. This is particularly important in areas where forage and grain legumes are cultivated on wetlands or temporarily flooded areas. Legume species differ markedly in adaptation to flood-prone areas (Striker and Colmer, 2017). Tolerant legume species are generally able to sustain the oxygen (O_2_) diffusion path under waterlogging via physiological adaptation. An increased aerenchyma network in the root and nodule cortex, the presence of a barrier to radial O_2_ loss in the outer root tissues and an increased permeability of the nodule O_2_ diffusion barrier (ODB) can facilitate tolerance (Striker and Colmer, 2017). Metabolic acclimation and the presence of alternative nodulation strategies are additional adaptation responses to waterlogging (Roberts et al., 2010).

The aim of this mini review is to explore the mechanisms underlying legume plant adaptation, symbiosis development and nodule functioning under waterlogging.

## Waterlogging Effects on Plant-Bacteria Interaction

### Effects of Hypoxia on Nodulation

Successful symbiosis involves an initial cross-talk between plants and bacteria, with the coordinated expression of genes from both partners to induce molecular re-programming, which leads to the development of a nodule (Oldroyd and Downie, 2008). Bacteria sense the plant-derived flavonoids of the root exudates and produce nodulation factors (named Nod factors), lipochito-oligosaccharide molecules that participate in bacterial infection and, when perceived by the plant, trigger the nodule’s specific developmental program (Dénarié and Cullimore, 1993).

Several studies have considered the waterlogging effect on nodulation capacity. Hypoxia-sensitive legumes, such as pea (Minchin and Pate, 1975), alfalfa (Arrese-Igor et al., 1993), and soybean (Sung, 1993) exhibit reduced nodule weight when grown under hypoxic conditions. *Medicago truncatula* nodulation shows a 45% decrease under 0.1 % O_2_ but is not affected by 4.5% O_2_ treatment, and the nodule fresh weight per plant is not dampened by 4 weeks of hypoxia (El Msehli et al., 2016). Two studies analyzing nodulation ratings of 21 species of annual pasture legumes and 13 species of perennial legumes (Nichols et al., 2008a,b) report that most legume, including waterlogging sensitive species such as *Melilotus albus* and *Medicago sativa*, showed effective nodulation after several weeks of inundation. In this context, it is unclear whether the nature of nodule types may support different mechanisms of dealing with the stress, considering that indeterminate nodules (*Medicago* spp., *Pisum* spp., and *Melilotus* spp.) are characterized by a persistent meristem and a continuous growth, while determinate nodules (*Glycine* spp, *Vigna* spp, and *Lotus* spp.) are characterized by a not persistent meristem and a limited growth potential.

In flood-tolerant legume species, the nodulation process shows some morphological and physiological adaptations. In *Melilotus siculus*, nodules formed during waterlogging stress have been observed above all on adventitious roots (Konnerup et al., 2018). Under flooding, *Sesbania rostrata*, a tropical legume that grows in temporary flooded habitats (Capoen et al., 2010), switches from a typical root hair curling (RHC) mechanism of nodulation to a lateral root based (LRB) one (D’Haeze et al., 2000; Goormachtig et al., 2004). When grown in aerated soils, *S. rostrata* nodulation occurs through the mechanism of RHC, where bacterial colony is entrapped in growing root hairs that start to curl. When LRB infection occurs, bacteria enter at the base of the adventitious or lateral roots where they form an infection pocket prior to bacteria release into the nodule primordium.

Interestingly, *S. rostrata* LRB nodulation requires ethylene (Goormachtig et al., 2004), whose production is stimulated in plants by flooding and accumulates under water due to a slow diffusion. Ethylene inhibitors blocks *S. rostrata* initiation of nodulation, since bacterial invasion, infection pocket formation and nodule primordia were not observed in hydroponic roots (D’Haeze et al., 2003). Moreover, ethylene is likely involved together with ROS in inducing the programmed cell death of cortical cells, which is necessary for the formation of the infection pocket occurring during crack invasion (D’Haeze et al., 2003).

On the other hand, ethylene accumulation inhibits the RHC invasion of *S. rostrata* (Goormachtig et al., 2004). The application of ethylene biosynthesis inhibitors resulted in an increased RHC nodulation, while the opposite was observed adding ethylene precursors (Goormachtig et al., 2004). Indeed, ethylene inhibits nodulation in several legumes, such as *M. truncatula* (Penmetsa and Cook, 1997) and *Pisum sativum* (Guinel and Sloetjes, 2000).

### Effects of Oxygen Availability on Nodule Functioning

Once inside the forming nodule, bacteria differentiate into bacteroids, which can fix N_2_ via the activity of nitrogenase enzyme, representing the fundamental reaction of the symbiosis (Roberts et al., 2010). Nitrogenase is inactivated by free O_2_, thus N_2_ fixation is made possible thanks to the microoxic conditions predominant in the nodules. Furthermore, bacterial genes for nitrogenase assembly are expressed at low O_2_ concentration (Soupène et al., 1995). Nodules have evolved adaptations to maintain an inner low O_2_ environment, among which the presence of the ODB and by expressing O_2_-carrying symbiotic plant hemoglobins (Appleby, 1992; Berger et al., 2018). Thus, the developing nodule shifts from a normoxic state during the formation of the symbiosis to a microoxic one in mature nodules (Witty and Minchin, 1990). As a consequence, nodules are naturally microoxic organs that maintain a low O_2_ level, while preserving an active energy production.

The presence of a flexible ODB that regulates the O_2_ influx into the infected zone of the nodule was questioned over years. The ODB is likely composed by cortical boundary layers, matrix glycoproteins and endodermis modifications, which depend on the nature of the legume-rhizobia association (Minchin et al., 2008). Early studies on nodule structure identified the absence of a physical barrier in the soybean nodules cortex and the presence of continuous air pathways (Bergersen and Goodchild, 1972; Sprent, 1972). Subsequently, studies on pea and lupine nodules identified few intercellular spaces in the cortical cell layers and the absence of intercellular space connections within the nodule infected areas (Dixon et al., 1981). Indeed, occlusion in intercellular spaces were observed in the inner cortex of soybean nodule exposed to high O_2_ level, suggesting the presence of a flexible mechanism of morphological and structural adaptation (Serraj et al., 1995).

As underground organs, nodules can be exposed to flooding. The adaptation of functioning nodules to waterlogging includes structural and metabolic changes. Several adaptive processes have been described in nodules, including the tight regulation of the ODB flexibility, the development of aerenchyma and the setup of a specific ATP regenerating metabolism under low O_2_ level. Hypoxia-tolerant *Lotus uliginosus* nodules under flooding showed a lower concentration of matrix glycoproteins within intercellular spaces of the cortex in comparison with the sensitive species *L. corniculatus* (James and Crawford, 1998). This suggests a hypoxia-dependent mechanism capable to decrease the occlusions under low O_2_ availability and finalized to open air pathways when necessary. Recently, nodules of *M. truncatula* exposed to high O_2_ concentration showed a tightening of the ODB (Avenhaus et al., 2016). As consequence, the modulation of the O_2_ supply to the infected zone may be a key factor of nodule activity regulation. Under high O_2_ concentration, after a transient nitrogenase inhibition, the recovery of nitrogenase was observed and attributed to flexible ODB (Hunt et al., 1989; del Castillo et al., 1992; Avenhaus et al., 2016).

A crucial trait for plant survival under waterlogging is the possibility to develop aerenchyma, in order to provide a path for O_2_ diffusion along the roots from the aerated organs above (Colmer and Voesenek, 2009). The fact that some forage legumes are sensitive to waterlogging has been attributed to the limited possibility of O_2_ flux through aerenchyma to the root nodules (Arrese-Igor et al., 1993; Pugh et al., 1995; Konnerup et al., 2018). Some tolerant legumes have developed an extensive network of aerenchyma tissues, as indicated by the tolerant species phenotype identified in Table 1.

**Table 1:** Waterlogging tolerant and sensitive legumes.

Species	Treatment	Phenotype	References
*Cicer arietinum, Vicia faba* (sensitive)	Deoxygenated stagnant solution (7 days)	Death of root tips	Munir et al., 2019
*Melilotus siculus* accessions (tolerant)	Deoxygenated stagnant solution (7 days)	Root phellem abundance	Striker et al., 2019
*Lotus tenuis, L. tenuis* ×*L. corniculatus* (tolerant)	Partial submergence stress (55 days)	Aerenchyma and adventitious root formation	Antonelli et al., 2018
*Melilotus siculus* (tolerant)	Waterlogging (21 days)	Aerenchymatous phellem in hypocotyl, roots and the outer tissue layers of nodules	Konnerup et al., 2018
*Pisum sativum* (tolerant accessions)	Waterlogging (4, 8 days)	Successful germination	Zaman et al., 2018
*Phaseolus vulgaris* (sensitive and tolerant accessions)	Flooding conditions (1, 10 days)	Root weight and germination rate traits associated to flooding tolerance	Soltani et al., 2017
*Lens culinaris* (sensitive and tolerant genotypes)	Waterlogging (6 days)	Successful germination	Wiraguna et al., 2017
*Vicia faba* (tolerant), *Pisum sativum* (sensitive), *Lupinus albus* (sensitive)	Waterlogging at flowering (0, 5, 10, 15, 20 days)	Better seed yield and biomass of shoots, roots and nodules in tolerant genotypes	Pampana et al., 2016
*Phaseolus coccineus* (tolerant)	Flooding (24, 48 hours)	Vascular cavity formation	Takahashi et al., 2016
*Pisum sativum, Lens culinaris* and *Lathyrus sativus* (sensitive and tolerant genotypes)	Waterlogging (14 days)	High root porosity and unaffected shoot nitrogen content in tolerant genotypes	Malik et al., 2015
*Melilotus siculus* accessions (tolerant)	Hypoxic saline condition (21 days)	Plant ability to regulate ions	Striker et al., 2015
*Aeschynomene americana* (tolerant)	Waterlogging (30–40 days)	High nitrogenase activity and growth	Tobisa et al., 2014
*Lotus japonicus* recombinant inbred lines (tolerant)	Waterlogging (21 days)	Aerenchyma formation and high stomatal conductance	Striker et al., 2014
*Melilotus siculus* (tolerant accessions), *Trifolium michelianum* (sensitive), and *Medicago polymorpha* (sensitive)	Waterlogging combined to salinity (5 days)	High root porosity in tolerant genotypes	Teakle et al., 2012
*Melilotus siculus* (tolerant)	Stagnant solution (21 days)	Aerenchymatous phellem development	Teakle et al., 2011
*Lotus tenuis* (tolerant)	Waterlogging (30 days)	Shoot elongation	Manzur et al., 2009
*Vigna radiata* (tolerant and sensitive genotypes)	Waterlogging (4, 8 days)	Availability of root sugar reserves in tolerant genotypes	Sairam et al., 2009
*Lotus* spp (tolerant and sensitive genotypes)	Waterlogging (19 weeks)	Aerenchyma and adventitious roots formation in tolerant genotypes	Real et al., 2008
Faba bean, yellow lupin, grass pea, narrow-leafed lupin, chickpea, lentil, field pea (tolerant and sensitive genotypes)	Waterlogging (7 days)	Adventitious root and aerenchyma formation in tolerant genotypes	Solaiman et al., 2007
*Lupinus luteus* (tolerant), *L. angustifolius* (sensitive) reciprocal- and self-grafted combinations	Waterlogging (14 days)	Tolerance influenced by the root genotype	Davies et al., 2000
*Trifolium tomentosum* (tolerant) and *T. glomeratum* (sensitive)	Hypoxic solution (7–21 days)	High root porosity in the tolerant genotype	Gibberd et al., 1999

Given that N_2_ fixation is sensitive to low O_2_ condition occurring under flooding, soybean nodules have shown an impaired N_2_ fixation activity when transferred to a hydroponic solution (Justino and Sodek, 2013; Souza et al., 2016). Under these conditions, a change in N metabolism (Souza et al., 2016) and in the export of N_2_ fixation products in the xylem have been observed (Amarante and Sodek, 2006). In soybean nodules under flooding, a reduction in asparagine an accumulation of γ-aminobutyric acid (GABA) has been detected, which have been suggested to have a temporary storage role (Souza et al., 2016). These changes were reversible during recovery. Under hypoxia, the activation of the alanine metabolism was observed in waterlogging tolerant *L. japonicus* root and nodules, independently of the N status of the plant (Rocha et al., 2010b). Alanine accumulation was also observed in soybean roots under waterlogging (Rocha et al., 2010a). Alanine metabolism may be crucial to prevent pyruvate accumulation in order to facilitate glycolysis during waterlogging (Rocha et al., 2010b).

A further adaptive mechanism is related to the presence of hemoglobin-like proteins in the nodules, recently renamed phytoglobins (Hill et al., 2016). Three types of phytoglobins (phytoglobin1, leghemoglobin, and phytoglobin3) have been characterized in legume nodules (Bustos-Sanmamed et al., 2011; Berger et al., 2018). They are known to buffer O_2_ concentration and to scavenge nitric oxide (NO). Hypoxia generates NO in plants, likely with the presence of a cyclic respiration that improves the plant’s capacity to tolerate hypoxic stress by maintaining the cell energy status (Igamberdiev and Hill, 2009; Gupta and Igamberdiev, 2011). This phytoglobin-NO respiration (PNR) involves the following phases: nitrate to nitrite reduction via the activity of nitrate reductase; nitrite translocation from the cytosol into the mitochondria; production of NO through the reduction of nitrite at both the cytochrome C oxidase and the alternative oxidase sites of the mitochondrial electron transport chain, which allows ATP regeneration; NO movement from the mitochondrial matrix to the cytosol; and NO oxidation to nitrate by phytoglobins.

Interestingly, functional nodules of *M. truncatula* (Baudouin et al., 2006), *Glycine max* (Meakin et al., 2007), and *L. japonicus* (Shimoda et al., 2009), have been shown to produce NO, and flooding conditions significantly increases NO production in soybean (Meakin et al., 2007; Sánchez et al., 2010), and *M. truncatula* hypoxic nodules (Horchani et al., 2011). In *M. truncatula* nodules, energy status appears to be dependent on the PNR cycle partly under normoxia and totally under hypoxia (Horchani et al., 2011). Thus, the functioning of PNR in microoxic nodules enables the plant to oxidize NADH and to sustain ATP synthesis also under O_2_ shortage.

## Oxygen Signaling in Plant and Bacterial Partners

### Oxygen Sensing in the Plant Partner

**FIGURE 1:**
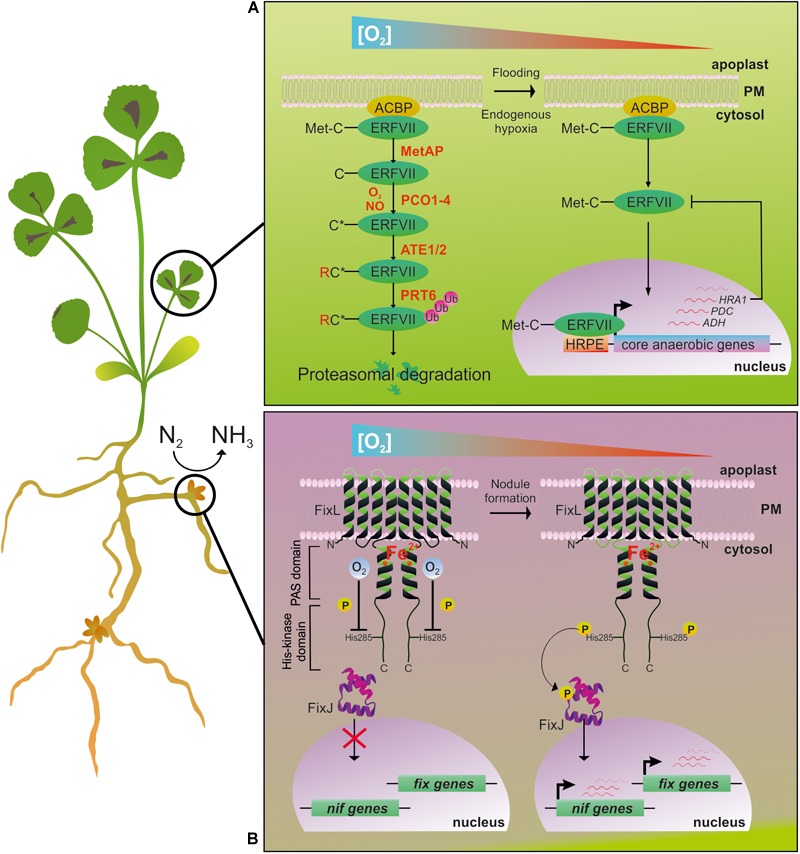
The main O_2_-sensing pathways described in plants (identified in *Arabidopsis* and hypothesized to be present in *M. truncatula*) and *S. meliloti* N_2_-fixing bacteria. **(A)** In *Arabidopsis*, the Cys branch of the N-end rule pathway for protein degradation allows the O_2_-dependent regulation of gene expression (Licausi et al., 2013). ERF-VIIs are a class of transcription factors characterized by a conserved N-termini (N-degron) in which Cys_2_ determines the protein’s fate in response to O_2_ level inside the cell. In aerobic conditions (**left**), ERF-VIIs are unable to activate the transcription of anaerobic genes. In these conditions, Met Aminopeptidase (MetAP) removes the N-terminal Met, and PCOs oxidize the resulting exposed Cys (C^∗^) (Weits et al., 2014; White et al., 2017). After arginylation by Arginyl Transferases (ATE1-2), an Ubiquitin Ligase (PRT6) identifies the proteins as a degradation substrate for the 26S proteasome. Under O_2_ deficient conditions (**right**), the efficiency of ERF-VIIs oxidation is dampened, allowing the stabilization and translocation into the nucleus to finally induce a set of anaerobic genes (Kosmacz et al., 2015), with Arabidopsis RAP2.2 and RAP2.12 playing a major role in comparison to the other ERF-VIIs (Bui et al., 2015). This also happens through fine regulation controlled by the Hypoxia Response Attenuator (HRA1), which antagonizes RAP2.12 through a feedback mechanism that enables a flexible response to different levels of O_2_ availability (Giuntoli et al., 2014, 2017). The *cis*-regulatory element Hypoxia Responsive Promoter Element (HRPE) has been identified as being enriched in some hypoxia-responsive genes (Gasch et al., 2016). **(B)** FixL-FixJ two-component regulatory system in *S. meliloti* symbiotic bacteria regulates the expression of *nif* and *fix* gene clusters in an O_2_-dependent way. In free-living bacteria (**left**), FixL is inhibited by the binding of O_2_ to the heme moiety inside the PAS domain. By establishing symbiosis with the plant, nodule formation gives rise to a microoxic environment surrounding the microbial cells (**right**). In turn, FixL is activated by auto-phosphorylation and transfers the phosphoryl group to the FixJ transcriptional activator, thus regulating *nif* and *fix* genes expression.

The Ethylene Responsive Factor group VII family (ERF-VII) guides the response to O_2_ level variations to ensure plant survival (Gibbs et al., 2011; Licausi et al., 2011). In *Arabidopsis*, this family is composed of five transcription factors which all possess an N-terminal amino acid (N-degron) and Cys residue in the second position of the protein. ERF-VII proteins are degraded via the N-end rule-dependent proteasome pathway triggered by Plant Cysteine Oxidases (PCOs) in an O_2_-dependent manner (Weits et al., 2014; White et al., 2017; Figure 1A).

Together with O_2_, NO destabilizes ERF-VIIs, and a reduction in the availability of either gasses is sufficient to stabilize them (Gibbs et al., 2014). The discovery of this O_2_/NO sensing mechanism has opened up new possibilities for better understanding the plant adaptation to low O_2_ and for improving flooding tolerance in crops.

An interesting link has been found between *Arabidopsis* ERF-VIIs and microorganisms. Infection by the obligate biotroph *Plasmodiophora brassicae*, which causes clubroot development (Gravot et al., 2016), was found to involve ERF-VIIs control. Subsequent to the identification of fermentation-related genes induced in infected root galls, the authors suggested that N-end rule-driven hypoxia responses are a general trait of pathogen-induced gall growth (Gravot et al., 2016). In the context of pathogenesis, the resistance to the hemibiotrophic pathogen, *Pseudomonas syringae* pv tomato has been shown to involve ERF-VIIs substrates to regulate pathogen-induced stomatal closure in Arabidopsis (Vicente et al., 2018).

To date, no data are available on the ERF-VIIs role in N_2_-fixing symbioses in legumes. In fact, the genome of *M. truncatula* (version Mt4.0^1^) harbors four genes that belong to the ERF-VIIs group (Boscari et al. (2013), personal communication), and phylogenetic analysis revealed the presence of ERF-VIIs in the *G. max* genome (Licausi et al., 2011). These ERF-VIIs harbor the conserved N-terminal degron, which suggests their control by O_2_ levels. A previous RNA-Seq analysis of *M. truncatula* during the symbiotic interaction with *Sinorhizobium meliloti* showed that *ERF-VII* genes are expressed in both roots and nodules (Boscari et al., 2013), where they may be crucial under microoxic conditions. ERF-VIIs might be an excellent candidate for deciphering O_2_ perception and NO signaling in N_2_-fixing symbioses. Indeed, interesting aspects are related to the possible targets of ERF-VIIs in nodule, which may be involved in morphological and metabolic adaptations in the microoxic nodule niche and under environmental hypoxia. In particular, speculation can be done on the possible role of ERF-VIIs on the metabolic modification in order to supply ATP under O_2_ scarcity and on the regulation of the ODB flexibility to different O_2_ level. Furthermore, it would be of interest to understand whether ERF-VIIs nodule targets may be involved in plant interaction with bacteria during the infection and the N fixation process.

### The FixL-FixJ Bacterial Two Component System

In N_2_-fixing rhizobia, the nitrogenase expression needs to be tightly regulated in response to changing O_2_ concentrations, due to the fact that O_2_ irreversibly inhibits the enzyme activity (Poole and Hill, 1997). The fine-tuning of nitrogenase related genes expression and the compartmentalization of the enzyme inside the nodule are thus prerequisites for an efficient N_2_ fixation (Soupène et al., 1995).

The induction of the N_2_-fixing gene cluster in *S. meliloti* and other symbiotic bacteria is regulated by a two-component system composed of the O_2_-sensing histidine kinase FixL and the response transcriptional regulator FixJ (Figure 1B; De Philip et al., 1990; Bobik et al., 2006). In *S. meliloti*, FixL is a protein composed of four transmembrane helices and a cytoplasmic region comprising a heme-containing Per Arnt Sim (PAS) domain and a C-terminal histidine kinase domain (Monson et al., 1992). The O_2_ sensing relies on the PAS domain (Gilles-Gonzalez, 2001), which is a widespread sequence found in bacterial (Green and Paget, 2004), animal (Adaixo et al., 2013), and plant (Christie et al., 2002) proteins. Oxygen exerts a negative regulation on FixL through interaction with the PAS domain.

The formation of a microoxic environment hampers the inhibitions that O_2_ exerts on FixL, and activates the reversible autophosphorylation of a His residue in the FixL kinase domain. Phosphorylated FixL transfers the phosphoryl group to the signal transducer, FixJ, whose phosphorylation status induces the transcription of the *nif* and *fix* gene clusters involved in nitrogen fixation and respiration (Reyrat et al., 1993; Bobik et al., 2006), via the activation of two intermediary regulatory genes, *nifA* and *fixK*. Interestingly, in *S. meliloti*, Meilhoc et al. (2010) identified about 100 genes up-regulated by NO, among which 70% have been described to be induced by microoxia (Bobik et al., 2006) and regulated through the FixL-FixJ system. NO present in nodules could serve as a signal to activate the FixL-FixJ system (Meilhoc et al., 2010).

## Concluding Remarks

The study of symbiotic models in response to waterlogging can help in deciphering the mechanism that may be crucial for the isolation of tolerant legume crop species and varieties in the field. The steps in signal exchange for the mutual recognition, nodule organogenesis and efficient N_2_ fixation under waterlogging are crucial aspects of the symbiosis. It would thus be of interest to decipher whether the sensing of O_2_ shortage in plant can (i) modify the perception of the partner during the symbiotic establishment, (ii) influence the nodule development, and (iii) affect the functioning of the nitrogenase enzyme in the bacteroid. These aspects may be further influenced by the high level of NO encountered in the nodule organ, which is involved, together with O_2_, in ERF-VIIs degradation. At the same time, the PNR cycle may offer an alternative way to produce energy under O_2_ shortage. A detailed analysis of these steps would help in finding interesting solutions for marginal land cultivation with waterlogging tolerant legumes capable of fixing N_2_ where limited O_2_ is available.

## Author Contributions

CP and AB conceived the idea of the review. All the authors were involved in the manuscript writing.

## Conflict of Interest Statement

The authors declare that the research was conducted in the absence of any commercial or financial relationships that could be construed as a potential conflict of interest.

## Footnotes

1 http://www.medicagogenome.org/home
